# Dr. Asa W. Jayne

**Published:** 1908-12

**Authors:** 


### OBITUARY

Dr. Asa W. Jayne died at his residence in North Tonawanda, November 16, 1908, aged 51 years, after a year's illness. Dr. Jayne was born in Norwich, and had practised medicine at North Tonawanda for the last 21 years. He was graduated from Hamilton College and from the Hahnemann Medical College at Philadelphia. Dr. Jayne was coroner of the first Niagara district, having been appointed by Governor Flower to succeed Dr. C. C. Smith, resigned. Besides a widow, two sons, Dr. Luther Jayne of La Salle and Arthur L. Jayne of N. Tonawanda, survive.

